# Metabolomic Profile of *Salicornia perennis* Plant’s Organs under Diverse *In Situ* Stress: The Ria de Aveiro Salt Marshes Case

**DOI:** 10.3390/metabo13020280

**Published:** 2023-02-15

**Authors:** Natasha N. Magni, Ana C. S. Veríssimo, Helena Silva, Diana C. G. A. Pinto

**Affiliations:** 1 LAQV-REQUIMTE, Department of Chemistry, Campus Universitário de Santiago, University of Aveiro, 3810-193 Aveiro, Portugal; 2 CESAM, Department of Biology, Campus Universitário de Santiago, University of Aveiro, 3810-193 Aveiro, Portugal

**Keywords:** *Salicornia perennis*, specialized metabolites, sugars, fatty acids, sterols, salinity, oxidative stress, TCA cycle, lipid peroxidation, pathogens attack

## Abstract

*Salicornia perennis* is a halophyte belonging to the botanical subfamily Salicornioideae that forms extensive perennial salt marsh patches. This subfamily has excellent potential, still unexplored, as a source of food, medicine, and phytoremediation. This study aimed to evaluate the lipophilic composition of the *Salicornia perennis* different organs inhabiting salt marshes of Ria de Aveiro under different stress regimes. For this purpose, the lipophilic content was extracted with hexane and subsequent GC-MS analysis of the extracts for each plant organ, which was collected in three different salt marshes of the Ria de Aveiro. High sugar content was detected in the stems, whereas in fruiting articles, the higher content was in fatty acids. Shorter-chain organic acids were concentrated in the stems and vegetative articles; waxes were detected in greater quantity in photosynthetic organs. More or less stressful environments induce changes in the ratio and composition of molecules, such as acclimatization and oxidative stress reduction strategies; for example, fatty acid content was higher in plants subjected to a higher stress regime. These data contribute to understand the metabolic pathways of the species under study, suggesting new research approaches to its potential as food, medicine, and phytoremediator.

## 1. Introduction

Salt marsh plants, designed by halophytes, are characterized by their ability to live and complete their life cycle in saline environments [1,2], exposed to at least 200 mM NaCl [3]. Plants, organisms without locomotion, are inevitably subject to a range of biotic and abiotic stressors, such as pathogen attacks, soil salinity, drought, nutrient concentrations, temperature changes, UV radiation, flooding, and pollution [4,5,6,7,8]. Adverse environmental conditions can cause drastic changes in plant cells’ chemical composition and physical state [9,10]. Stress, mainly abiotic [11,12,13,14,15,16], leads to a more significant amount of reactive oxygen species (ROS). These molecules are by-products of normal aerobic metabolism but can also be increased due to adverse conditions [5]. Plants generate ROS through electron leakage from metabolic activities in mitochondria, chloroplasts, peroxisomes, or other cellular compartments, such as the endoplasmic reticulum, plasma membrane, cell wall, and vacuole [5,17].

ROS act as cellular signals in small concentrations, regulating essential processes such as photosynthesis and stress signals [8]. However, as the concentration increases, damage also begins to occur, and plants, especially halophytes, must develop adaptations to survive stressful environmental conditions. These adaptations largely depend on the balance between ROS production and detoxification by the action of the cellular antioxidant machinery [5]. ROS accumulation disrupts redox homeostasis, resulting in lipid peroxidation, nucleic acid oxidation, and decreased photosynthetic capacity, causing carbohydrate damage, protein/enzyme inactivation, and triggering programmed cell death [17,18].

To survive and thrive in adverse conditions, halophytes have evolved mechanisms to adapt to these edaphic factors [4,6,7], one of which is the use of antioxidants that can be enzymes or specialized metabolites. One of the most harmful free radicals, ^•^OH, cannot be eliminated enzymatically, depending on high concentrations of antioxidants [5]. Stress tolerance depends on gene expression, antioxidant content modification, and the activity of these antioxidants. Antioxidant molecules can be polysaccharides, sugar alcohols, organic acids, phenolic compounds, and tocopherol [5,19]. Antioxidants can prevent the formation of free radicals, sequester them, or promote their degradation [19].

Salt affects the osmotic balance of plants, inducing their cells to lose water, ionic toxicity, nutritional imbalance, oxidative stress, metabolic disturbance, membrane disorganization, genotoxicity, and reduced cell division and expansion [20]. Osmotic regulation, in turn, is an essential process of adaptation to adverse environmental conditions, such as drought and salinity, and aims to maintain cellular turgor and protect cellular components. These osmotic adjustments are accomplished by accumulating osmotically active substances called osmolytes. They accumulate on the cytoplasm in response to these environmental stresses and protect the cellular machinery from denaturation, preventing dehydration and maintaining the fluidity and stability of the plasma membrane, guaranteeing balance on the osmotic potential and cellular homeostasis [3,21]. They are osmotically compatible and do not interfere with normal cytoplasmic functions [3,13,15]. Examples of osmolytes are substances such as sterols, phenolic compounds, amino acids, polysaccharides, and glycosides [3,13].

In addition to salinity, halophytes from coastal environments also experience the effects of inundation or waterlogging [3]. Exposure to anoxic conditions and transition from anoxia to re-oxygenation implies an imbalance in cellular redox potential, also involving in production of ROS [22], which leads to cell damage, oxidation of biological molecules, metabolic disorders, and senescence processes [2,11,16]. Plants have a variety of mechanisms to deal with flooding conditions and, therefore, with oxidative stress, including a series of metabolic responses at the anatomical and cellular levels. At the cellular level, there is the preservation of energy and reduction of respiration, which increases the availability of soluble sugars, in addition to the synthesis of antioxidant compounds, such as tocopherol, phenolic compounds, and others, to eliminate ROS and ensure cell integrity [11,13,22].

Low-intensity stress usually stimulates acclimatization metabolic responses. When stress is more intense, and the acclimatization threshold is extrapolated, defensive mechanisms are triggered to combat oxidative stress [8,10,21,23]. Therefore, the outcome of the environmental challenge depends on the delicate balance between ROS production and elimination mechanisms, as well as the intensity and duration of the stress [5,24].

Amaranthaceae (syn. Chenopodiaceae) is a botany family with an incredible biodiversity of halophytes [3,25]. These species have many biological activities, suggesting great pharmacological, biotechnological, and food potential [26,27]. The species under study belong to this family, commonly known as “perennial glasswort”, “perennial marsh samphire”, or “sea asparagus” in the *Salicornia* genus [28]. *Salicornia perennis* Mill. (syn. *Sarcocornia perennis* subsp. *perennis*) [29] vegetate on extensive areas of Ria de Aveiro’s salt marsh. They are small prostrate to erect subshrubs (20 cm high) with radicant low woodiness stems. The photosynthetic portion comprises distinct succulent and articulated segments in vegetative or in flowering/fruiting phenological state at its apices. The perennial habit distinguishes these species from the annual species of *Salicornia*. In the Iberian Peninsula, it is found in Huelva, the central and southern coasts of Portugal, and the Cantabrian-Atlantic coast. It always occurs in salt marshes reached by tides, which are limited to the low zones of salt marsh [30,31,32,33].

The scarcity of data about the perennial species of *Salicornia*, and the wide variation in chemical composition due to adaptations to stress conditions, guided the interest in the present study, which aims to evaluate the lipophilic composition of *Salicornia perennis*, detect and quantify its phytochemical composition, evaluate possible differences between plant’s organs and locations with different environmental conditions, and analyze changes in the metabolomic profile.

## 2. Materials and Methods

### 2.1. Study Area

**Figure 1:**
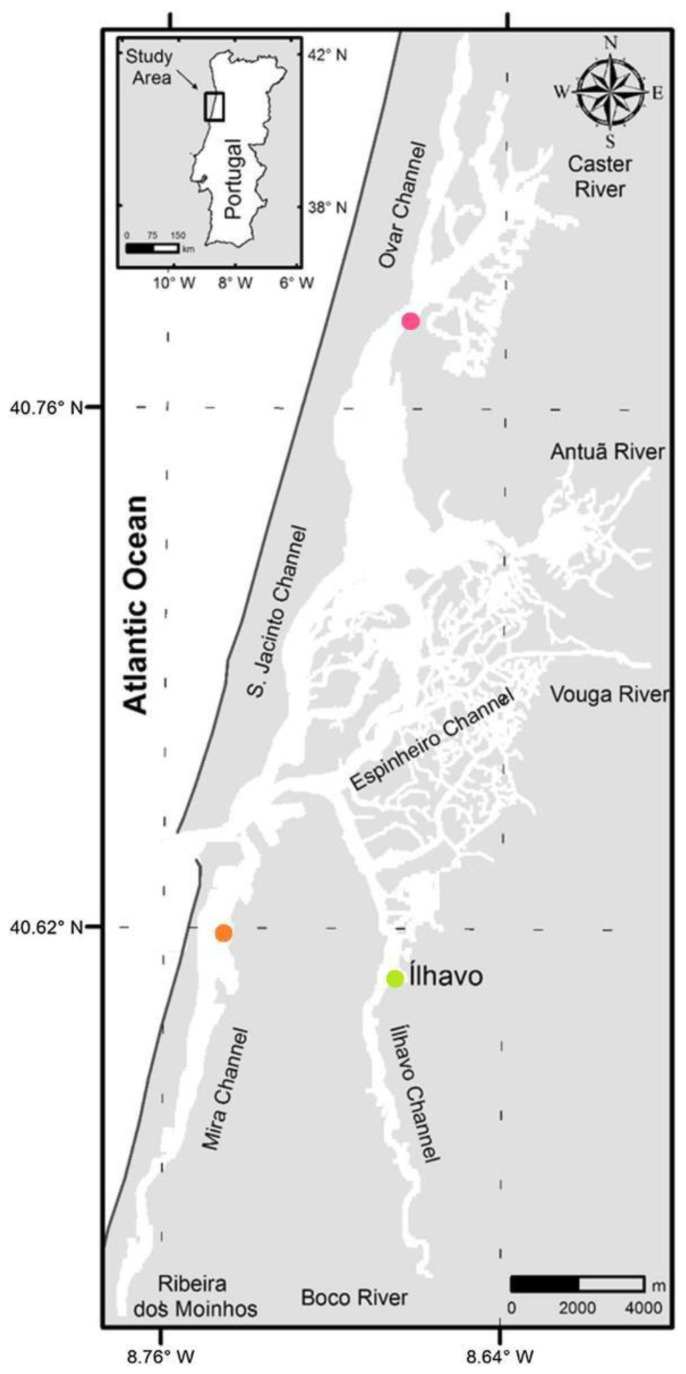
Ria de Aveiro with the location of the three sampling sites: Mira salt marsh (orange dot), São Jacinto salt marsh (pink dot), and Ílhavo salt marsh (green dot).

The Ria de Aveiro (Figure 1) is a coastal lagoon situated on the Northwest Atlantic coast of Portugal, separated from the sea by a sand spit. The lagoon’s dynamics are mainly forced by semi-diurnal tides, with the tidal range varying between 0.6 m at neap tides and 3.6 m at spring tides, with an average of about 2 m [34,35]. Three sampling sites were chosen to collect the plants and the respective environmental conditions: Ílhavo channel salt marsh, Mira channel salt marsh, and São Jacinto channel salt marsh. All the studied salt marshes are inundated twice a day by the tide.

### 2.2. Sample Data

Before sample collecting, taxonomical identification of the species was performed in the field. Sampling material was harvested in low water in September 2021 to collect samples from the same plant’s patch in all phenological states: vegetative and flowering/fruiting. In each sample, the aerial portion of the plant and sediment of the underground where the plant vegetates were collected. Samples were stored in plastic bags and transported to the laboratory in refrigerated boxes.

### 2.3. Environmental Conditions

#### 2.3.1. Salinity

Determination of salinity was performed on the laboratory. Briefly, 30 mL of distilled water was added to 10 g of previously dry sediment. This mixture was stirred (Nahita Magnetic Stirrer Model 690/5) at 100 rpm for 2 h and 30 min. After the sample had rested for 2 h, salinity was recorded (‰) using a WTW LF 196 salinometer.

#### 2.3.2. Waterlogging

To determine the water content of the sediment, 50 g of fresh sediment was dried in an oven at 105 °C, until obtaining a constant weight. The difference between fresh weight and dry weight is precisely the water content, expressed as a percentage of the fresh sediment. Information about the tidal flooding patterns of the sampled marshes was provided by the Estuarine and Coastal Modelling Division (NMEC), and was obtained through the output analysis of a hydrodynamic model applied to the Ria de Aveiro.

### 2.4. Plant Preparation

**Figure 2:**
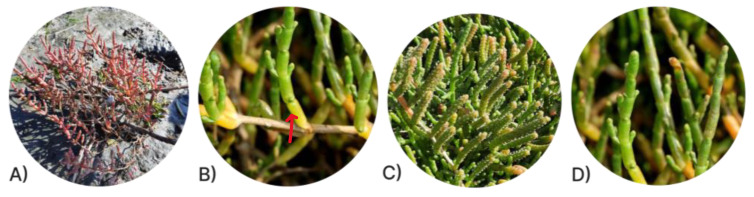
*Salicornia perennis*. (**A**) Complete plant; (**B**) Stems (red arrow); (**C**) Fruiting segments; (**D**) Vegetative segments.

The aerial portions of the collected plants were washed in running water, at the end passed in distilled water, and divided into woody portions (stems) and fleshy portions (segments). The segments were subdivided into vegetative and fruiting segments (Figure 2). Before extraction, the samples were dried in an oven at 60 °C for two days and the resulting material was cut into small pieces and powdered using an electrical blender.

#### Plant Extract Preparation

Briefly, 10 g of plant powder was determined on a precision scale (precision of d = 0.1 g) from each plant’s organs (stems, vegetative segments, and fruiting segments) obtained for each sampling site. Each portion of the plant had three replicates, totalizing 27 samples of plant’s extracts. The samples were extracted with the solvent hexane (2 g:30 mL), at room temperature, in Erlenmeyer flasks sealed with aluminium foil and parafilm to prevent evaporation and avoid the influence of light on the decomposition of some compounds. A magnetic stirrer bar was added to the flask and both were placed on a stirring plate for 72 h at 600 rpm.

**Figure 3:**
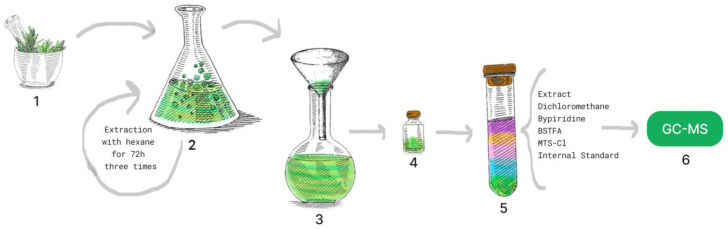
Schematic representation of the extraction steps for further analysis. 1. Powdered plants; 2. Powdered plants + hexane + magnetic stirring for 72 h; 3. Filtering of the solvent; 4. Drying of the extract on the rotary evaporator; 5. Extract silylation; 6. Analysis in GC-MS.

The extraction was repeated three times, after each extraction the solvent was renewed, and the extraction continued for another 72 h. The solvent from the combined extractions with hexane was filtered into a previously weighed flask and then evaporated to mass consistency on a Rotary Vacuum Evaporator, originating a plant residue extract (Figure 3).

### 2.5. Gas Chromatography-Mass Spectrometry Analysis

#### 2.5.1. Trimethylsilyl Derivatives Preparation and GC-MS Analysis

The extracts were analysed with Gas chromatography-=Mass Spectrometry Analysis (GC-MS), which three independent replicates of each extract were submitted to the silylation procedure—a total of 54 silylated extracts—and each one was injected in duplicate—a sum of 108 chromatograms to be analysed. The silylation reaction consists of converting the present compounds’ hydroxyl and/or carboxyl groups into trimethylsilyl (TMS) ethers and/or esters. Making these compounds volatile and readable by the GC-MS column [36,37].

Dichloromethane (645 µL) was added as a solvent to 10 mg of each sample, then dissolved on an ultra-sonic bath. For the silylation, 125 µL of pyridine 125 µL of BSTFA (bis(trimethylsilyl)trifluoroacetamide) and 25 µL of TMS-Cl (trimethylchlorosilane) were added. Finally, 80 µL of internal standard C36 (hexatriacontane) solution was added, totalizing 1 mL. The mixture was kept at 70 °C for 30 min, then cooled for a further 30 min, filtered, and added to the vial for analysis (Figure 3).

GC-MS analysis of each silylated sample was performed using a GC-MS QP2010 Ultra Shimadzu (Universidade de Aveiro, Portugal) equipped with a ZB-5ms capillary column (30 m × 0.25 mm i.d. and a film thickness of 0.25 μm). Samples were injected with a split ratio of 10.0 and helium as carrier gas with a flux of 1.17 mL/min. The column temperature was maintained at 90 °C for 4 min and then increased to 16 °C/min until 180 °C, followed by 6 °C/min until 250 °C and lastly 3 °C/min until it achieved 300 °C, which was maintained for 5 min. The injector temperature was 320 °C, and the transfer-line temperature was 200 °C. The mass spectrometer operated in the electronic impact (EI) mode with an energy of 0.1 kV. Data were collected at a rate of 1 scan/s over a range of *m*/*z* 50–1000. The performed chromatography lasted 52.96 min. The compounds were identified based on a direct 21 comparison with the mass spectra database libraries (NIST14 Mass Spectra and WILEY Registry TM of Mass Spectra Data).

#### 2.5.2. Compound’s Identification and Quantification

For quantitative analysis, calibration curves were obtained with pure reference compounds at different concentrations. These compounds were chosen to represent the main extract components (glycerol, 1-eicosanol, oleic acid, palmitic acid, cholesterol, β-sitosterol, succinic acid, and galactose). The standards were injected in at least 6 different concentrations (15–300 µg/mL) and a fixed amount of internal standard (hexatriacontane). Different standard concentrations were chosen to ensure that the concentration of each compound in the extracts was obtained by interpolation. Limits of detection (LOD = 3 × standard deviation/slope) and quantification (LOQ = 10 × standard deviation/slope) were calculated. Results were expressed in mg of compound/g of extract, as mean values of the 3 extract replicates and their respective chromatograms (2 readings each).

### 2.6. Standards and Reagents

Several pure compounds were used as standards to ensure the identification of the phytochemicals and to perform the calibration curves for quantification purposes: glycerol (99%), 1-eicosanol (99%), oleic acid (99%), palmitic acid (99%), cholesterol (99%); β-sitosterol (99%), succinic acid (99%), palmitin (99%), galactose (99%). For extraction, hexane (≥95%) (Fisher chemical) was used for the extracts, while dichloromethane was employed to dissolve the extracts before silylation. Pyridine, *N*,*O*-bis(trimethylsilyl)trifluoroacetamide (BSTFA) (99%) and trimethylsilyl chloride (TMSCl) (99%) (Sigma-Aldrich, Schnelldorf, Germany) were applied in the sample’s derivatization by silylation.

Results were expressed in mg of compound/g of extract, as mean values of the 3 extract replicates and their respective chromatograms (2 readings each).

### 2.7. Statistical Analysis

Three replicates of each plant organ were performed, for each sampling site, 2 replicates of each silylated extract, and 2 reading replicates by GC-MS. This means that each sample originated 12 chromatograms (e.g., for stems from Ílhavo). Only compounds detected in >75% of replicates were considered. These compounds were quantified in the 12 chromatograms and used to calculate the standard deviation. Principal Component Analysis (PCA) were performed, after normalising the data, for environmental and phytochemical data. All the data were performed on the statistics software PRIMER (version 7). The data recorded as the mean ± standard deviation (m ± SD) were submitted to analysis of variance (ANOVA) followed by a post hoc honestly significant difference (HSD) Tukey’s test at *p* < 0.05.

## 3. Results and Discussion

### 3.1. Statistical Data

**Figure 4:**
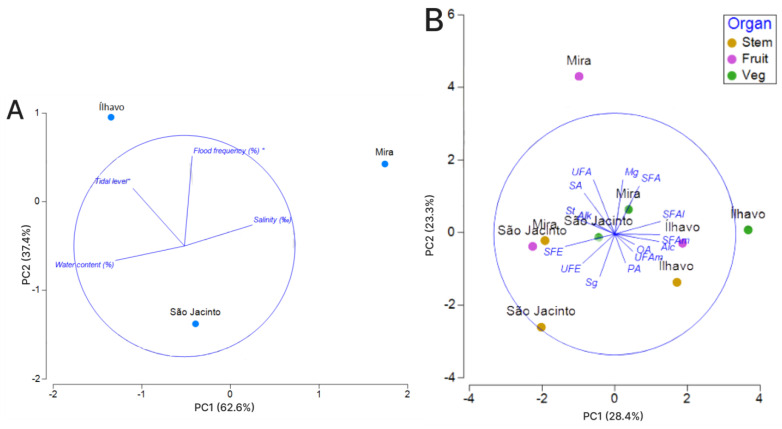
Principal Component Analysis (PCA) ordination diagram for environmental data (**A**) and for families of compounds (**B**). (**A**). Distribution of sedimentary conditions by location. (**B**). Distribution of phytochemical data by family of compounds (Alc = alcohols; Alk = alkanes; OA = organic acids; Mg = Monoacylglycerols; SFA = saturated fatty acids; SFE = saturated fatty esters; SA = sugar alcohol; UFAl = unsaturated fatty alcohol; UFE = unsaturated fatty esters; SFAl = saturated fatty alcohols; SFAm = saturated fatty amides; SFE = saturated fatty esters; St = sterols; Sg = sugars; UFA = unsaturated fatty acids; UFAm = unsaturated fatty amide), location, and plant’s organ. * Data provided by NMEC.

For the statistical interpretation of the environmental data, a Principal Component Analysis (PCA) was carried out (Figure 4A; Table 1). The PCA revealed that, concerning the sediment conditions, the variance of the data is relatively median; however, Ílhavo and São Jacinto share some characteristics, such as salinity and water content, which are in the negative portion of the PC1 axis (62% of the variance) but differ in waterlogging conditions, such as tidal level and flood frequency. The PC2 axis is responsible for 37.4% of the variance; Ílhavo and Mira share the positive portion, with the waterlogging variables being the most representative. Mira is the site with the most challenging sedimentary conditions, quite different from the other two sites, its shares waterlogging conditions with Ílhavo. Still, salinity is the most representative variable in this location.

For the statistical interpretation of the families of compounds data, a Principal Component Analysis (PCA) was also carried out (Figure 4B; Table 1). The PCA for the families of compounds revealed that samples from Ílhavo and Mira have the most different compound content, Ílhavo occupying the positive portion of the diagram in relation to the PC1 axis and Mira occupying the negative portion. São Jacinto presents a more intermediate position concerning the PC1 axis, and this axis explains 27.2% of the variation. Regarding the PC2 axis, the negative portion is dominated by the stems, whose variance is explained by the sugar content (Sg), which differs considerably from the other organs of the plant. The positive portion of the PC2 axis is occupied by fleshy segments, which are quite different between fruiting and vegetative segments and between sampling sites. Particular emphasis is given to the fruited segments of Mira, which differed considerably in the content of unsaturated fatty acids (UFA), and to the vegetative segments of Ílhavo, with a higher content of saturated and unsaturated fatty amides (saturated fatty alcohols) and waxes.

### 3.2. Environmental Data

Environmental data are shown in Table 2. The Tidal Level determines how often the plant in question will be bathed by the tide, according to the height of the marsh where the plant is located. Together with the water content and flood frequency, they give us an idea of the waterlogging to which these plants are subjected.

According to the information provided by NMEC about tidal inundation for the sampling sites, on the Tidal Level, all sampled plants are flooded by the tide twice a day all year round—MHW: mean high water, flooded all year round; MHWN: mean high water neaps, flooded all year round. Ílhavo salt marsh reveals the highest levels of Inundation Frequency (70.98%). Contrastingly, São Jacinto salt marsh shows the smallest values for this variable (46.85%). Water content was much lower in Mira salt marsh (32.56%) than in Ílhavo (64.27%) or São Jacinto (59.34%). Regarding salinity, Mira salt marsh presented substantially higher values (34.0‰), and Ílhavo and São Jacinto had lower and similar salinities (18.5‰ and 19.73‰).

### 3.3. Phytochemical Data

**Figure 5:**
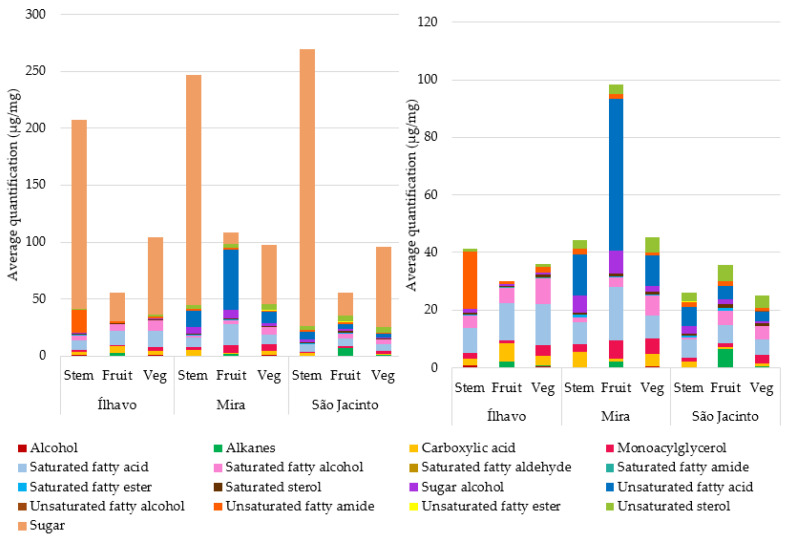
Mean values of phytochemical composition (µg/mg) of *Salicornia perennis*, with the respective contribution of each family of compounds, divided by salt marsh, and the organs of the plant (Stem; Fruit: fruited segments; Veg: vegetative segments). On the left with the presence of sugars, and on the right subtracting the contribution of sugars.

The lipophilic profile of *S. perennis* for the different organs (stems and vegetative/fruiting segments) was analysed for the three sampling locations. A total of 103 compounds were identified, of which 79 were quantified (Table 3, Table 4, Table 5, Table 6, Table 7 and Table 8) and 24 as non-quantifiable compounds (Table S1), either because they were present in trace amounts or because the necessary standards were unavailable. The identified lipophilic compounds were distributed into 17 classes, whose amounts relative to each sampling site and plant’s organs can be compared simultaneously in Figure 5 and in quantitative detail in Table 3, Table 4, Table 5, Table 6, Table 7 and Table 8.

#### 3.3.1. Sugars

Figure 5 shows the relative average chemical profile subdivided into groups of compounds, sampling sites, and plant organs. It is possible to perceive that different plant organs have different compositions. The most abundant group of compounds was the sugars (Figure 5 and Table 3).

In Figure 5, the presence of sugars for all samples is evident but more expressive in stems. Carbohydrates are primary products of photosynthesis and provide energy to produce and sustain plants’ biomass. In addition, they serve as signallers of transcriptional processes [21]. Disaccharides accumulate when growth is inhibited in light-stress situations, but photosynthesis is not [5]. At the cellular level, the preservation of energy and reduction of respiration takes place, which increases the availability of soluble sugars, in addition to the synthesis of antioxidant compounds, such as tocopherol, phenolic compounds, and others, to eliminate ROS and ensure cell integrity [11,13,22].

Sugars have a dual role concerning ROS; on the one hand, the metabolism of sugars during photosynthesis is a source of ROS. However, sugars also act as cellular signallers, and variations in their content can signal responses to osmotic stress, decreasing the cell’s ROS production while acting as antioxidants, interacting with them, and scavenging free radicals. In addition, they can be osmoprotective molecules, ensuring cell turgor and, thus, stabilizing membranes [5,21].

The production and accumulation of soluble sugars such as sucrose, fructose, and glucose are a response to stressful environmental conditions such as salinity, drought, low temperature, and flooding [21]. The highest concentrations of sugars were found in the stems of *S. perennis*, which, as it is a low-marsh species, is subjected to a regime of seawater submersion twice a day in the Ria de Aveiro.

The salinity in Mira is the highest, together with the lowest water content in the sediment, making Mira salt marsh with the highest osmotic stress. This species is entirely adapted to waterlogged and salinity conditions but is more sensitive to drought [38,39]. Hence, its physiological response concerning the synthesis of sugars is more intense due to this balance between moments of drought and waterlogging twice a day in the three locations. This can be compared with the amount of sugars in São Jacinto. In this case, the species is subjected to the less limiting conditions: the lowest salinities and waterlogging. As an inhabitant of the low marshes, *S. perennis* is subject to flooding twice a day, even in marshes where the frequency of flooding is lower, as in São Jacinto. For this reason, the synthesis of sugars is more pronounced in the stems of this species and remains elevated even with slightly lower salinities in response to twice-daily flooding.

The higher concentration of sugar in the roots is strategic. The vascular bundles of the adventitious roots, and consequently the stems, are the gateway for water and solutes through the plant’s xylem. Therefore, they must be capable of osmotic adjustment to develop in saline environments, such as reverse osmosis, when cells surrounding the xylem lower their osmotic potential by the accumulation of sugars, increase turgor pressure, and absorb water against osmotic potential [40]. The transporting of sugars from the photosynthetic organs to the other parts of the plant also contributes to increasing sugar content in stems since the products of photosynthesis are transported via the phloem. Salt stress increases the demand for ATP, raising the synthesis of sugars needed to supply this demand [41]. Regarding the types of sugars found (Table 3), it should be highlighted that the GC-MS analysis performed did not aim to detect sugars, so it is possible to perceive sugars and their quantity but not the exact identification. Table 3 shows the most likely sugars.

Sucrose is a disaccharide that combines fructose and glucose units. It is the principal sugar in a plant’s metabolism and is vital in stress acclimatization [21]. This molecule can act as an osmolyte, maintaining the turgor pressure of cells, preventing desiccation, and protecting proteins from denaturation, in addition to acting as a signaling agent [5]. Sucrose has free radical scavenging activity superior to monosaccharides, as it has more OH groups to interact with ROS [21].

Salinity triggers a physiological drought in plants generated by low osmotic potential, ionic and nutritional imbalance, or a cumulative of all these factors. To overcome this adversity, the cell concentrates sugars as compatible solutes, increasing proportionally with the salinity [21]. The sample with the highest sucrose concentration was the stems from São Jacinto, a sampling site with less limiting sedimentary conditions. Although the accumulation of sugars is correlated with adaptation to stressful conditions, such as increased salinity, ensuring osmoregulation, these metabolic responses also have higher energy costs. Therefore, the higher concentration of sugars in São Jacinto may indicate a photosynthetic increase due to milder conditions or that these sugars were less spent on acclimatization responses to stress and were than accumulated [41].

Secondly, plants from Mira show the highest values for sucrose. The sugar content may be due to the high salinity combined with high waterlogging and the low water content in the sediment. The concentration of sugars is directly related to the water content in the plants. The hydroxyl groups retain the water molecules maintaining membrane integrity and preventing protein denaturation [21]. The high energy demand of the physiological responses necessary for acclimatization may have consumed some sugar reserves when photosynthesis is reduced [41]. Plants from Ílhavo and São Jacinto salt marshes were subject to similar environmental conditions; however, plants from Ílhavo are subject to higher waterlogging frequency pressure, which is reflected in their sugar content.

#### 3.3.2. Fatty Acids

In second place are fatty acids (Figure 5), with the presence of saturated fatty acids (SFA) evident in all studied samples, with slightly different concentrations for each sampling site and plant organ. Unsaturated fatty acids (UFA) are abundant in Mira and absent in Ílhavo, and in São Jacinto, they are found in concentrations slightly similar to SFA ones. Monoacylglycerols are reserve lipids and were relatively more abundant in Ílhavo and Mira.

Fatty acids (FA) are long-chain carboxylic acids, indispensable as energy sources and nutrients for plant survival and growth [42]. In addition to reserve molecules, they are essential components of membranes, present in many physiological processes [42]. The relative proportions of the constituent lipid classes determine the fluidity and permeability of membranes. Changes in these proportions and changes in the fatty acid residue saturation and sterols lead to changes in the membrane’s physical and chemical properties. These modifications respond to environmental, biotic, or abiotic changes [10,43,44].

**Figure 6:**
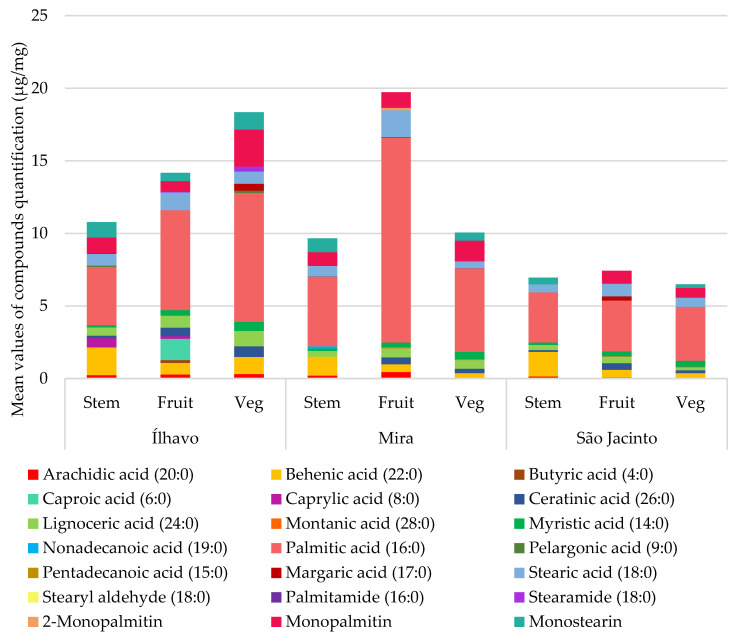
Average total composition of saturated fatty acids (µg/mg), with the respective contribution of each compound, in each salt marsh, and plant’s part (Stem; Fruit: fruited segments; Veg: vegetative segments).

Figure 6 shows the relative quantities of saturated fatty acids (SFA) and the monoacylglycerols of saturated fatty acids and SFA derivatives for each site and part of the plant, quantified in Table 4. The highest values of saturated fatty acids were found in Ílhavo and Mira, locations with the highest waterlogging conditions. Whereas plants from the São Jacinto salt marsh, located in a place with the lowest waterlogging conditions, have intermediate composition and concentration of compounds.

**Figure 7:**
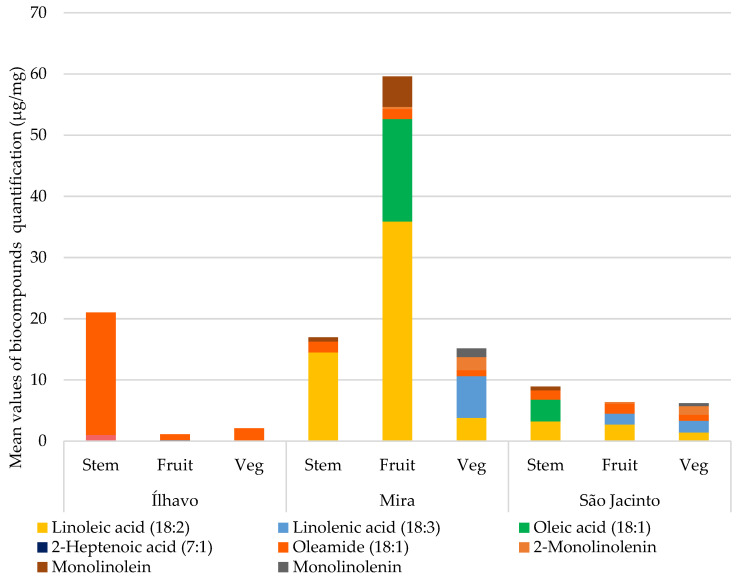
Average total composition of unsaturated fatty acids (µg/mg), with the respective contribution of each compound, by salt marsh, and plant’s organ (Stem; Fruit: fruited segments; Veg: vegetative segments).

Figure 7 compares the chemical species of unsaturated fatty acids (UFA) and the monoacylglycerol conjugates of unsaturated fatty acids and UFA derivatives from each plant’s organs for each sampling site, quantified in Table 4. The highest concentration of unsaturated fatty acids was found in Mira. In Ílhavo, UFAs are practically absent, except for oleamide, a derivative of oleic acid, which has anti-inflammatory [45] and antimicrobial activity [46,47] and shows strong inhibitory effects against some pathogenic bacteria [47]. Oleamide appears in similar and lower concentrations in Mira and São Jacinto but in a very high concentration in Ílhavo. Plants from São Jacinto have a lower concentration for UFA but similar to the one found for SFA.

Fatty acids do not usually remain in a free state in the cell. On the contrary, they are usually stored in the form of triglycerides (TG), diacylglycerols (DG), monoacylglycerols (MG) or conjugated with other molecules. Triglycerides, along with their more hydrolysed forms, are neutral lipids and constitute the leading reserves of fatty acids for energy production and carbohydrate synthesis [48]. In a situation of energy deficiency, fatty acids are recruited to undergo β-oxidation. Then, TG breaks down, reducing them to DG and releasing a fatty acid; the DG is broken down into MG, releasing another fatty acid residue. Finally, the last break of MG resulting one last fatty acid molecule and one glycerol [49]. These released fatty acids from β-oxidation are again lysed into acetyl- CoA molecules, which are incorporated into the Glyoxylate Cycle, becoming an alternative source of succinic acid for the Krebs Cycle (see further section Organic Acids). In the Krebs Cycle, this succinic acid is converted into malic acid, which in turn is converted into glucose, and, finally, sucrose, supplying the cell’s energy needs. The β-oxidation reaction is regulated by the soluble sugars concentration and is inhibited when the concentration of sugars is increased [50,51].

**Figure 8:**
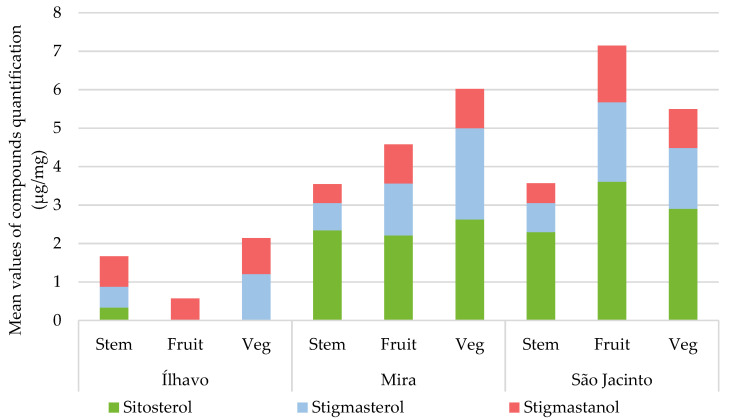
Average total composition of sterols (µg/mg), with the respective contribution of each compound, divided by salt marsh, and part of the plant (Stem; Fruit: fruited segments; Veg: vegetative segments).

The analysis performed, using extraction of the plant material with hexane, and followed by silylation of the extract before the GC-MS analysis, is not the most recommended to understand if the fatty acids present in the lipophilic profile were initially free, being mobilized for β-oxidation or if they were conjugated with glycerol in triglycerides, diacylglycerols or monoacylglycerols. The amount of glycerol (Figure 8) and the fact that high concentrations of free fatty acids are toxic and induce cell death [52] suggest that they were conjugated.

Given the reduced amount of saturated and unsaturated fatty acids and the higher concentration of sucrose in *S. perennis* from São Jacinto, these plants may undergo β-oxidation to supply their carbohydrate needs and maintain osmotic potential and cellular metabolism under salt stress. However, this location has the highest sugar concentration and the least stressful sedimentary conditions at the time of harvest, which does not justify the need for β-oxidation.

The production of reactive oxygen species (ROS) is a continuous process. It is part of a regular plant’s metabolism, especially in halophytes. Still, the excess can cause oxidative damage to the entire cell, in this case, to lipids and membrane integrity, in a process called lipid peroxidation [43,53]. The most reactive ROS is ^•^OH, it can interact with each molecule in its vicinity, causing damage to molecules and impairing physiological processes. Therefore, stressed cells benefit from higher concentrations of ^•^OH scavengers. In contact with fatty acids, ^•^OH can initiate membrane lipid peroxidation, in which ROS interact with double bonds of fatty acids, making them progressively more saturated, in a chain reaction that affects membrane fluidity and integrity [5,53]. Lipid peroxidation occurs in plants exposed to various abiotic stresses, such as salinity, drought, submersion, and metal exposure [5]. Fatty acid remodelling under stress normally occurs to stabilize membranes and maintain cellular integrity, thus, a higher concentration of unsaturated fatty acids has the physiological objective of increasing the fluidity and permeability of the plasma membrane, in turn, higher saturation leads to greater rigidity and impermeability [54]. 

Lipid peroxidation is part of the physiological processes of acclimatization of plants, especially halophytes, which are usually exposed to stressful environmental conditions. However, species have acclimatization thresholds to oxidative stress [5], and even halophytes can experience conditions beyond their physiological limits when the plant undergoes oxidative stress.

In the lower Mira salt marsh, *S. perennis* experiences the most stressful environment among those sampled, with high salinity and waterlogging. The high content of saturated and unsaturated lipids, combined with the highest amount of glycerol and monoacylglycerols among the plants, gives an indication but is not quantifiable of the high concentration of reserve lipids. In addition, the high sugar content, and the lower content of fatty acids in comparison to Ílhavo, indicates that the plants are under acclimatization, with some β-oxidation, especially in stems and vegetative segments. The results suggest no lipid peroxidation and the rise of plasma membrane fluidity due to the presence of UFA.

There is a particularly unexpected result in Ílhavo. Sedimentary conditions are similar to those found in the São Jacinto salt marsh, except for the waterlogging, however Mira shows the same flood conditions. One would expect the lipophilic profile to be identical regarding the richness and diversity of compounds, and intermediate to the other two locations [38,39]. However, these plants have the higher lipid peroxidation, with the absence of unsaturated fatty acids, a condition of increased oxidative stress. Sugars and saturated fatty acids are elevated, so β-oxidation does not appear to occur.

When lipid peroxidation occurs, polyunsaturated lipids progressively become monounsaturated and later saturated. Linolenic acid (18:3) has its unsaturation decreased, becoming linoleic acid (18:2), which in turn is converted into linoleic acid (18:2); if the ROS stimulus continues, it is converted into oleic acid (18:1). Still in stearic acid (18:0), and finally palmitic acid (16:0) [55]. Thus, we can see that, except for Mira, the saturation of fatty acids—their conversion into increasingly saturated molecules—is greater than the unsaturation. This is because the saline environment to which halophytes are subjected has an expressive presence of ROS, and more stability is required in membranes. Therefore, Mira being the location with the highest salinity, it is unexpected that it would contain the highest concentrations of UFA.

#### 3.3.3. Sterols

Saturated sterols are present in all samples, for all plant organs, generally in less quantity in stems. On the other hand, unsaturated sterols are absent or almost absent in the samples from Ílhavo and appear more expressively than saturated sterols in the other samples.

Phytosterols are essential components of the cell membrane and lipid rafts, involved in plant growth and development, playing a crucial role in various physiological and biochemical processes, and consequently contributing to biological functions during development and resistance to stress on plants [56]. Phytosterols are synthesized by the post-squalane pathway, which divides into two branches, one responsible for the synthesis of terpenes and the other of sterols, both crucial for the plant’s responses to abiotic stress [56,57]. They can occur in two forms: free or conjugated with esters, glycosides, and fatty acids [10,57].

As integral components of the membrane, sterols maintain the integrity, fluidity, and permeability of the lipid bilayer, thus increasing the stress resistance of plants. In addition, a plant’s sterols form lipid rafts, a unique structure that helps establish cell polarities, signals, and plant-pathogen interactions, growth, and development, such as seed germination, plant’s phenotype, senescence, timing flowering and seed yield [56,57].

The sterol profile can change in response to environmental stimuli, such as biotic and abiotic stresses. Sterols conjugated with fatty acids can be found in all the plant’s tissues and used as a storage pool in times of scarcity [57]. The most abundant in plants are β-sitosterol, stigmasterol and campesterol. β-Sitosterol is the principal sterol that strengthens the plant’s cell membranes; its relationship with stigmasterol plays an essential role in the structure and function of cell membranes, varying the concentrations and proportions of the two compounds according to the stress response [10,57]. The relationship between these two sterols is critical for the plant’s growth and development [10,44].

It has been reported that sterols can also respond to water stress, with a significant increase in sterol content in plants exposed to water deprivation. This further implies that these compounds may play an important role in water stress tolerance, reinforcing membranes, and increasing rigidity [10,56]. Also, drought-tolerant plants have higher sterol content when exposed to water privation stress [9,10].

Sterols are also involved in the saline stress response [56], an example is the relative reduction in sitosterol and an increase in stigmasterol observed in *Brassica oleracea* roots exposed to salinity [56]. Due to increasing membrane resistance, the increase in sterol concentration indirectly influences the protection of membrane lipids. Treatment of *Capsicum annuum* leaves with sitosterol significantly offset damage from salt stress, such as electrolyte loss and inhibited growth, subsequently improving membrane stability and antioxidant activity. In *Spartina patens* subjected to salinity treatments, the most abundant lipid remained stable with increasing salinity in combination with an increased relative percentage of campesterol and decreasing sitosterol [56].

Phytosterols are necessary for the architecture of plants, where they affect the synthesis and transport of the plant’s hormone auxin. Plants with sterol deficiency may show dwarfism phenotype [56]. Phytosterols also mediate reproductive development, processes such as flowering time, stigma development, pollen formation, and seed generation can be seriously affected, resulting in malformations and partial sterility [56]. *Arabidopsis thaliana* with impaired β-sitosterol biosynthesis showed dwarf phenotype caused by insufficient cell elongation [44]. Organs linked to reproduction are also susceptible to changes in the sterol profile compared to stems [44].

However, all plant sterols influence and regulate plasma membrane fluidity with different efficiencies [44]. Added to the membrane’s saturated or unsaturated lipid composition impact, they result in other physicochemical properties [44]. Other lipids are much more transient and have more dynamic responses to any cellular effects and changes; sterols, in turn, are more stable and promote the ordering of membrane structural components, playing a considerable role in stress adaptation processes. β-sitosterol is the precursor of stigmasterol, and this is the stress sterol, as it is a more saturated version of the first one [44].

Figure 8 represents the sterol profile of *S. perennis* in the three sampling sites, with differentiation between the plant organs; quantification details are in Table 5. The phytosterols found were β-sitosterol, stigmasterol, and stigmastanol; the first two are unsaturated, and the third is saturated. Plants from Ílhavo showed an impressively low sterols content, considering that these compounds are essential for cell membrane cohesion. Stigmastanol appeared in all parts of the plant, stigmasterol was detected in stems and vegetative segments, and β-sitosterol only in stems. In the field, this plant presented a smaller phenotype than the other sampling sites, and finding fruited segments for the analysis was a challenge. The sterol deficit is undoubtedly affecting the plant’s fitness.

The plants from the other sampling sites did not show much variation in concentrations, with the stigmasterol content increasing in São Jacinto species, mainly in the fruiting articles and in the plants from Mira. This happens in vegetative articles. The place with the highest salinity is Mira, where plants have a relatively higher content of total sterols; however, São Jacinto has quite a similar sterol content and much milder environmental conditions.

#### 3.3.4. Alcohols

Alcohols were found in smaller amounts, nonetheless, with a great diversity of compounds, such as sugar and long-chain alcohol. These last ones are essential components of waxes, which, jointly with alkanes, are a part of the plant’s protection system against water excess or deficit.

**Figure 9:**
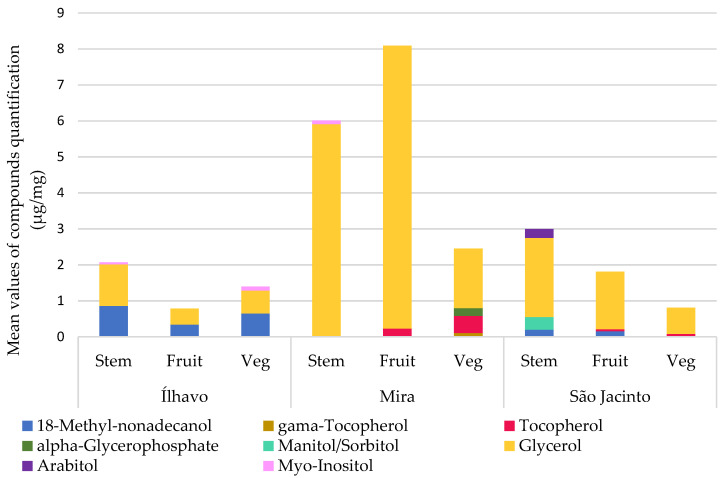
Average total composition of alcohols and sugar alcohols (µg/mg), with the respective contribution of each compound, divided by salt marsh, and part of the plant (Stem; Fruit: fruited segments; Veg: vegetative segments).

Figure 9 shows the relative concentrations of alcohols and polyols for each plant’s organs at the three sampling sites, details in Table 6. Sugar alcohols or polyols are derived from sugar-reducing metabolism. Their function in cells is as compatible osmolytes, the most common being glycerol, mannitol, and sorbitol. The concentration of sugar alcohol increases in response to osmotic stress, which can be caused by exposure to salinity or water deprivation. They mitigate oxidative stress, scavenge free radicals, and adapt to osmotic stress by maintaining cellular turgor and preventing macromolecules from inactivation [21,58].

Glycerol may be associated with the alternative synthesis of glucose for energy supply in the Krebs cycle, function as a compatible osmolyte, or combine with fatty acids to form lipid reserve molecules such as triglycerides [59]. However, the increase in glycerol concentration, often in conjunction with arabitol, is a hyperosmotic stress response where glycerol synthesis comes from glucose [59]. Furthermore, Dias et al. [35] found that a decrease in glucose content and an increase in sugar alcohols suggests that the pool of polyols is maintained at the expense of glucose. The polyols found in plants from Ílhavo have a small concentration of glycerol and myo-inositol. The highest glycerol concentrations were detected in Mira, as well as a reduction in glucose content; this species is well adapted to waterlogging [38,39] and is currently inhabiting the sediment with high variability between waterlogging and dryness, besides, it is the location with the highest salinity. So, the high glycerol content may be an acclimatization response to a combination of momentaneous water deficit (water content), waterlogging and high salinity, with glycerol acting as an osmolyte, or they can be conjugated with lipids as a reserve. In São Jacinto, plants have some amount of glycerol, plus mannitol/sorbitol, as the location with the least stressful sedimentary conditions, it would be expected to have a lower concentration of polyols than Ílhavo. The glycerol detected by the GC-MS could be in the form of osmolytes or conjugated with other storage molecules.

Inositols are a family of cyclohexyl alcohols, the most common of which is *myo*-inositol. They are widely distributed throughout the plant’s kingdom and are functionally necessary for normal growth and development, membrane biosynthesis, and signal precursors. Increased inositols are reported when species are subjected to salinity, desiccation, or UV radiation [8,21]. *Myo*-inositol was found in detectable amounts in stems and vegetative segments from Ílhavo and stems from Mira. In Mira, the conditions are lower in water content, and higher in salinity and waterlogging, so this could be the case of osmotic adjustment with inositols. In Ílhavo salt marsh, the conditions are similar to those found in São Jacinto, but with greater waterlogging, therefore the presence of *myo*-inositol, which is absent in São Jacinto.

Tocopherols are hydrophobic alcohols with high antioxidant activity. In plants, they are produced through the mevalonic acid pathway, the same route of synthesis of sterols, terpenes, and carotenoids. Tocopherols are responsible for eliminating reactive oxygen species, recycling via enzymatic pathways, and repeating the process. Thus, they play an essential role in response to biotic and abiotic stress [5,8]. Some stressful environmental conditions, such as increased UV-B radiation, salinity, drought, or waterlogging, stimulate tocopherol biosynthesis [56]. They function as antioxidants, inhibit the propagation of lipid peroxidation, and protect polyunsaturated fatty acids, preventing oxidative damage to membranes and lipoproteins [19,60]. Tocopherol also activates defence pathways, particularly those associated with pathogenesis [8].

Figure 9 and Table 6 show the alcohols and polyols detected for each plant’s organ at each sampling site. There are two compounds from the vitamin-E group, tocopherol and γ-tocopherol, absent in plants from Ílhavo. γ-tocopherol was only detected in the vegetative segments of *S. perennis* from the Mira salt marsh. In clearly higher amounts in Mira, tocopherol is already present in the fructified and vegetative segments from Mira and São Jacinto. This compound is not expected to appear in large quantities since it is not a cell constituent, as are fatty acids or sugars. However, its higher Mira content indicates these plants’ efficient antioxidant capacity under environmental stress. Mira lower salt marsh is the sampling site with the most challenging sedimentary conditions, so having a stock of antioxidants is an adaptive characteristic of halophytes [38,39]. In São Jacinto, tocopherol occurs in smaller amounts, and these are also the least stressful environments. Intriguingly Ílhavo, with the intermediate environment, conditions which have already shown an oxidative stress response in the profile of other compounds, such as fatty acids and sterols, do not present tocopherol, which indicates even more oxidative stress, disproportionate to the sedimentary characteristics to which the species is submitted.

#### 3.3.5. Waxes

**Figure 10:**
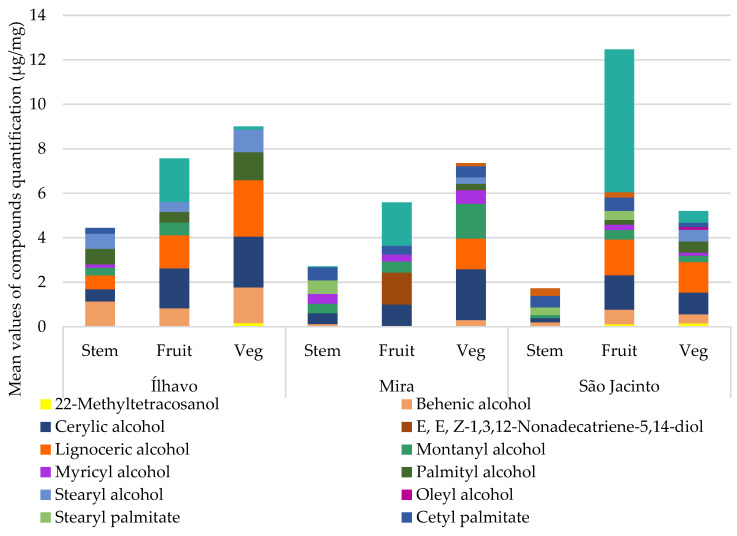
Average total composition of waxes (µg/mg), with the respective contribution of each compound, divided by salt marsh, and part of the plant (Stem; Fruit: fruited segments; Veg: vegetative segments).

Waxes are a heterogeneous mixture of lipophilic substances, mainly alkanes or esters of long-chain fatty acids (from 14 to 36 C) conjugated with long-chain alcohols (from 16 to 30 C), formed from the esterification reaction of two molecules into waxes. The silylation reaction can break this bond between the alcohol and the fatty acid, and the two are detected separately as shown in Figure 10.

The cuticle is a protective structure that covers the epidermis cells, and this layer of wax often covers these cells. Epicuticular wax is the first line of defense against adverse conditions and constitutes a physical-chemical protection barrier [61]. Some plants may increase the cuticular thickness of leaves, stems, and fruits in response to adverse environmental factors to protect themselves from radiation, prevent water loss, or protect against pathogens [62]. Plants in aquatic environments have very thin cuticles [10,35,63]; however, halophytes are not aquatic but amphibious plants, which may exhibit xeromorphic characteristics, such as cuticular thickening, similar to plants of drought conditions [64]. This factor prevents excess transpiration and water loss [65].

Figure 10 and Table 7 show the content of alkanes and fatty alcohols, which constitute the waxes, for each organ, at sampling sites. The wax content is substantially lower in the stems, except for Ílhavo Waxes are expected to cover more humid parts of the plant’s body, such as leaves, stems have some woodiness and lower water content where waxes are less efficient. The fructified and vegetative parts of the *Salicornia* species are very similar. The amphibian behaviour of these fleshy plants explains a well-developed cuticle in response to a salty and perhaps drought environment, which may need some barrier to help against the water loss in the photosynthetic segments.

The alkane content was higher in the fruiting articles of all samples but with an even greater emphasis on the São Jacinto plants, the sampling site with the lower salinity. Grossi & Raphel [63] refer to the higher alkane production than fatty acid esters, which agrees with our results. However, the same authors indicate that the reason for the greater production of alkanes to the detriment of the production of fatty acid esters is unknown.

#### 3.3.6. Other Organic Acids

Organic acids are more concentrated in all segments from Ílhavo, and stems and vegetative segments from Mira, with a significant amount in the stems from São Jacinto. This was a group of compounds with a particular diversity of compounds and considerable differences in concentration between sampling sites and plant’s organs.

**Figure 11:**
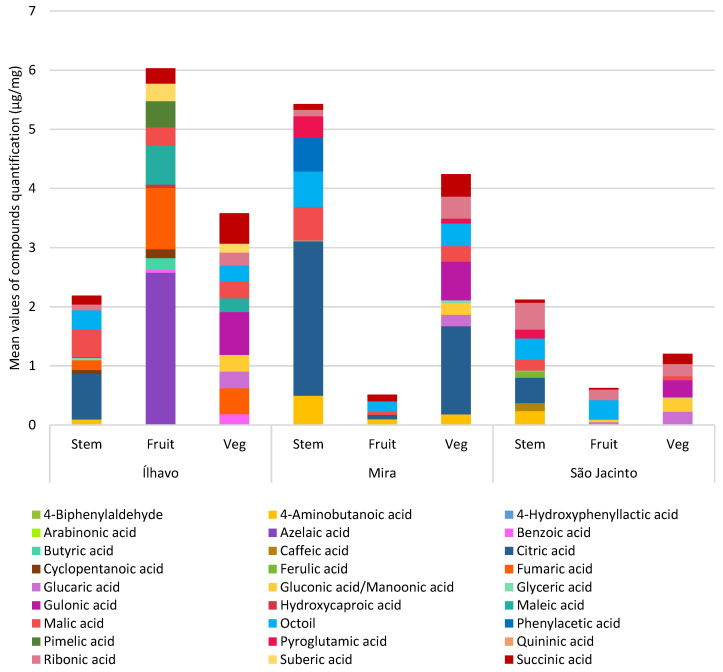
Average total composition of organic acids (µg/mg), with the respective contribution of each compound, divided by salt marsh, and part of the plant (Stem; Fruit: fruited segments; Veg: vegetative segments).

Figure 11 and Table 8 show the other organic acid quantification, which includes phenolic acids and excludes the previously discussed fatty acids for each plant organ at each sampling site. A remarkable diversity of organic acids was found to be quite variable between the organs of the plant and the sampling site. Ílhavo has a very different lipophilic profile, especially regarding these organic acids, compared to the other sampling sites.

The Tricarboxylic Acid Cycle (TCA) or Krebs Cycle is one of higher organisms’ main carbon metabolism pathways, essential for cellular respiration. The interruption of the flow of electrons in the TCA cycle stops a series of processes and syntheses, affecting the carbon pool of the entire cell [41]. Salt stress increases the demand for ATP to operate adaptive mechanisms such as ion homeostasis, defense against ROS, etc. Therefore, the ability to tolerate adverse conditions depends on the efficiency with which the plant faces its energy needs in the presence of the stress factor [41].

Citric, succinic, fumaric, and malic acids participate in the TCA cycle as intermediaries and do not accumulate under normal conditions but under certain stressful situations. Das Prabal et al. [41] also noticed an increase in malic, citric, and succinic acid in salinity-resistant rice cultivars and argue that this increase in carbon for the Krebs Cycle may indicate, in addition to antioxidant activity, an increase in energy production, even under saline stress. In this case, higher levels of organic acids represent the plant’s ability to survive and maintain growth even under stressful conditions. This is a characteristic metabolic response of halophytes. Glycophytes under saline stress show a decrease in organic acid content, which indicates an inhibitory effect caused by saline stress [41]. As most physiological studies are carried out with glycophytes such as *Arabidopsis thaliana*, these variations may go unnoticed [41].

Citric acid is the primary organic acid of the Krebs Cycle, and it starts the cycle. This acid accumulates under oxidative conditions. The concentration of citric acid was increased in cotton plants under water stress and alfalfa under salt stress [66]. In the more specific case of a halophyte, *Puccinellia tenuiflora* also accumulated citric acid under adverse alkalinity conditions [66].

The next of these organic acids to be synthesized by the Krebs Cycle is succinic acid. It continues the cycle and produces fumaric acid. However, in a condition where the accumulation of ROS inhibits the cycle, this acid can also accumulate [8]. Still in the TCA cycle, fumaric acid can give rise to maleic acid, which is its isomer, or to malic acid, which provides continuity to the cycle. Maleic acid is a product of the oxidation of fatty acids [8]. The enzyme that encodes the reaction of fumaric acid to malic acid is sensitive to oxidative stress, which can lead to an increase in the concentration of fumaric acid at the expense of malic acid [67]. This situation is expected under reduced respiratory activity or anoxia and may be associated with amino acid degradation by the Urea cycle [68]. Malic acid plays a central role in the plant’s metabolism as an intermediary in the TCA cycle and precursor of citric acid synthesis, restarting the cycle and in stomatal responses [8,69].

The highest concentration of citric acid was found in the stems of all sampled plants and vegetative segments from Mira. Furthermore, *S. perennis* from Mira also showed higher amounts of succinic acid and malic acid, which are also intermediates in the TCA cycle, so the plant seems to be acclimatizing in response to the challenging environmental conditions since this is the sediment with the lowest water content, and higher salinity and waterlogging. Surprisingly, the content of these organic acids was low in the fruiting segments.

*Salicornia perennis* from Ílhavo also showed high concentrations of citric acid in its stems and was the plant with the highest content of succinic, fumaric, and malic acids, intermediates in the TCA cycle. However, the concentration of citric acid was only detected in the stems. Maleic acid was also present in the fruiting and vegetative segments, which indicates that the cycle is at least partially inhibited. Part of the fumaric acid stores gives rise to maleic acid to the detriment of malic acid, which is the intermediary that promotes citric acid synthesis.

Under stressful conditions, glucose can be converted to gluconic acid rather than TCA cycle intermediates [8,67]. Gluconic acid is an organic acid whose increased concentration in tissues may indicate a blockage in the Pentose Phosphate pathway, which in turn will impair the biosynthesis of fatty acids and the maintenance of the redox potential against free radicals, in addition to promoting an accumulation of GABA. This can occur in the presence of an inadequate concentration of oxygen, below the minimum required for cellular respiratory processes [68].

Gluconic acid gives rise to pyroglutamic acid, which in turn becomes glutamic acid (or glutamate), and later proline. Proline is one of the most studied and documented compatible osmolytes in plants regarding osmotic balance under stress, maintenance of turgor pressure, ROS detoxification, and protecting membranes, proteins, and enzymes from denaturation [70,71]. Proline accumulation is thought to be protective during dehydration, cold, and salt stress [71,72].

4-Aminobutanoic acid (GABA) is synthesized under stressful conditions in response to hypoxia, excessive heat or cold, drought, injury, herbivory, or pathogens. This synthesis can occur in two ways: using proline or by decarboxylation of glutamate. GABA is a cytosolic pH regulator, a reserve of C or N, a signalling molecule in case of exposure to abiotic stress, and participating in stomatal closure [8,68,73].

In Ílhavo, gluconic acid and GABA were found in the stems, together with the accumulation of maleic acid, which may indicate a blockage in the Pentose Phosphate pathway due to oxidative stress. In Mira, gluconic acid was found in fruiting and vegetative articles, pyroglutamic acid in stems and vegetative segments, and GABA in all parts of the plant, in this case, probably in response to the higher salinity and waterlogging. São Jacinto showed pyroglutamic acid in stems and GABA in stems and fruiting articles, even with less stressful conditions, this plant is still subject to twice-daily flooding and salinity.

Under oxidative stress conditions, the Krebs Cycle can be partially inhibited. ROS can oxidize amino acids to supply respiratory substrates during stress [8]. Protein oxidation can serve as an alarm signal to initiate or propagate plants’ responses to abiotic stress. Among the amino acids most frequently found under stress are lysine, isoleucine, leucine, valine, and proline [8]. Their concentrations progressively increase with increasing water stress, together with sugars and polyphenols for *Salicornia brachiata*, with the aim of osmotic regulation, macromolecule protection, nitrogen storage, pH maintenance, and free radical scavenging [71].

The amino acids are shown in Table S1; due to the GC-MS derivatization conditions used in this work, the amino acids could not be quantified. However, the program provides a semiquantitative result that gives us some idea of the amount of amino acids present for comparison purposes. Amino acids appeared more expressively in Mira, particularly proline, which was detected in stems (0.13%) and vegetative segments (0.18%), in addition to valine and isoleucine. This same plant had gluconic acids, pyroglutamic, and GABA in its composition, which indicates the necessary osmotic adjustment responses under flood and salinity; however, the content of organic acids from the TCA cycle suggests that it is not inhibited. Proline was also detected in São Jacinto stems (0.04%), in a much smaller amount, in addition to valine, GABA, and pyroglutamic acid, indicating the mildest osmoregulation response, consistent with the less stressful environment. In the same way as Mira, there is no indication of inhibition of the TCA cycle by the presence of intermediate compounds of the pathway.

Phenolic acids are secondary metabolites that originate from primary metabolites, such as carbohydrates, amino acids, and lipids, intending to protect against environmental conditions, pathogens, or competition. They are responsible for many vegetables’ characteristic smell, color, and taste [9]. Phenolic compounds are mainly produced via the Shikimate pathway, including simple phenols, phenolic acids, flavonoids, coumarins, stilbenes, hydrolyzable or condensed tannins, lignans, and lignins [9]. Phenolic acids are produced from phenylalanine, and converted into cinnamic acid, which can form ferulic, caffeic, and benzoic acids, among other compounds. Benzoic acid gives rise to another synthesis pathway for salicylic acid and phenylpropanoids [9].

Phenolic acids have antioxidant activity, protecting plants against reactive oxygen species produced by normal metabolism but have increased synthesis under stress [9,15,74]. For this reason, the proportions of these compounds can be affected under biotic and abiotic stress conditions. There is an increase in the synthesis of phenolic compounds induced by salinity, mainly moderate [74], in addition to acting as protectors against UV radiation associated with cuticle thickening [10,75]. They sequester ROS through the reactivity of the phenol fraction, inhibit ROS synthesis, and have antimicrobial activity [9]. Phenolic acids also act in the absorption of nutrients, protein synthesis, structural components of the cell, enzymatic activity, photosynthesis, allelopathy, and as precursors of phenolic lipids [9].

Hydroxycinnamic acids, such as ferulic and caffeic acid, play an important role in protecting against oxidative stress conditions [9,57] and scavenging nitrogen dioxide radicals, a toxic compound from chemical fertilizers [6]. Ferulic acid occurs in the plant’s tissues, mainly as low-molecular-weight conjugates in the cytosol or bound to cell wall polymers, rarely in a free state [76]. This phenolic acid is involved in saline stress and osmotic stress tolerance mechanisms, strengthening the cell wall and cell elongation [77].

On the other hand, hydroxybenzoic acids, derived from benzoic acid, lead to the synthesis of several other phenolic compounds, including plant hormones, and co-factors [78], mainly polar, and therefore not detected by the GC-MS. The synthesis of benzoic acid can also be induced by pathogens [8,79].

Figure 11 and Table 8 show the average quantification of organic acids, for each plant’s organ, at each sampling site. The phenolic acids found were caffeic, ferulic, and benzoic. *S. perennis* from Ílhavo presented only benzoic acid, which, given the absence of other phenolic acids, may indicate biotic stress by pathogens [8]. Surprisingly, no phenolic acids were detected in Mira due to the high salinity. However, it is possible that they are being mobilized for other routes of synthesis of polar phenolic compounds. São Jacinto plants have caffeic and ferulic acid, which agrees with the salinity response, but contrasts with the result found in Mira.

The cleavage of fatty acids within the plant’s cells produces a series of by-products, which can provide us with information about the origin of this stress [80]. One of these by-products is azelaic acid, produced by the hydrolysis of C18 unsaturated fatty acids such as oleic, linoleic, and linolenic acids. These fatty acids are essential for the survival of plants, and lipid peroxidation can also occur due to abiotic stress; however, the concentration of azelaic acid present in plants under attack by pathogens is much higher than the basal level of this acid [81]. Noctor et al. [8] reiterate that the compound is not routinely detectable by GC-MS analysis, as it is present in low concentrations.

Azelaic acid is an organic dicarboxylic acid with multiple and diverse functions. On plants, this compound acted mainly as an inducer of the plant’s immunity and acquired resistance to pathogens. Previous studies with *Arabidopsis thaliana* and *Solanum lycopersicum* (tomato) also report this acid as a signaling molecule for infection by pathogens [81,82].

Only plants from Ílhavo presented azelaic acid, and at a remarkable concentration. This observation, complemented with other phytochemical results of this plant, namely phytosterol deficiency, low content of unsaturated fatty acids and tocopherol, high concentration of oleamide, and higher content of benzoic acid, justify its stress condition and its probable attack by pathogens.

#### 3.3.7. Phytochemical Responses to Stress

**Figure 12:**
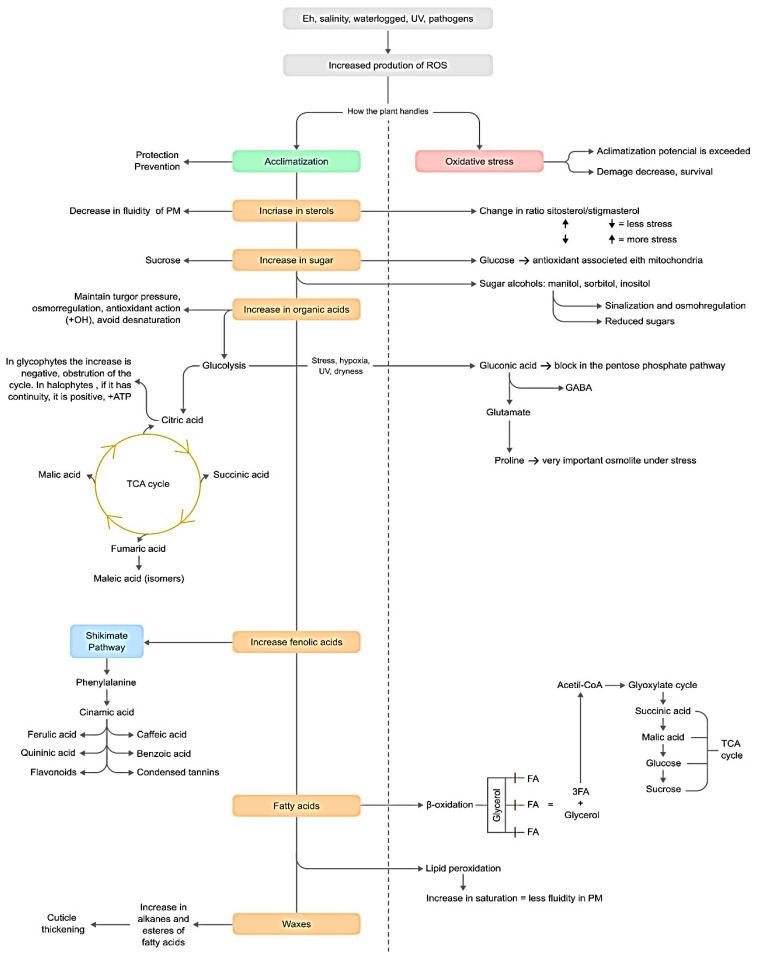
Synthesis processes of the main lipophilic compounds mediated by environmental stress. Eh = redox potential; UV = Ultraviolet; ROS = reactive oxygen species; ATP = adenosine triphosphate; TCA = tricarboxylic acid cycle; FA = fatty acids.

The phytochemical information, in general, provided valuable information about the response of this species to environmental conditions and how the content of these compounds is mediated according to physicochemical environmental characteristics. It is a complex process involving many variables, and halophytes face many environmental challenges simultaneously. Consequently, *S. perennis* is adapted to challenging environmental conditions. However, even halophytes have an acclimatization threshold and can be subject to oxidative stress from ROS accumulation when salinity, waterlogging, or drought becomes too intense. To deal with this stress, plants modify physicochemical and physiological properties by remodeling their phytochemical profiles. Changes in the ratio of saturated and unsaturated fatty acids can make the plasma membrane rapidly more or less fluid, and the sterol composition has the same purpose, but in the medium term. The accumulation of sugars is used to maintain the osmotic potentials and the turgor pressure of the cells; these sugars can be converted into polyols, signaling molecules, and inducing osmoregulatory responses. Other organic acids and phenolic compounds are used as antioxidant compounds, increasing in concentration as needed. Glycolysis feeds and initiates the Krebs Cycle and the cycle’s intermediate molecules, such as succinic, malic, fumaric, and citric acid. An increase in the content of only a few intermediates indicates obstruction of the cycle; however, an increase in all indicates high ATP synthesis, which is important for the high energy needs of acclimatization in halophytes. Cycle disruption can occur under stressful conditions and leads to an increase in proline, GABA, and gluconic acid. These processes are summarized in Figure 12.

In Ílhavo, *S. perennis* showed a different lipophilic profile than the other sampled plants. Despite being located in a salt marsh with a higher flood frequency, the remaining physicochemical characteristics of the sediment don’t seem to justify these differences, being the sampling location with the intermediate environmental conditions. It was possible to perceive that this plant was under environmental stress that may be induced by pathogens, revealing signs of high oxidative stress, such as intense lipid peroxidation, and the absence of essential molecules for antioxidant activity, such as tocopherol and phenolic acids. High sterol deficiency is critical to maintaining cellular integrity and efficient stress response. A high concentration of oleamide has inhibitory effects against some pathogens. The prominent presence of benzoic acid and azelaic acid indicates a pathogens attack. The plants were under a level of starvation, showing a reduced habit and absence of floral/fruiting segments in the flowering season.

In Mira salt marsh, *S. perennis* is subject to the most stressful environmental conditions, high salinity and waterlogging, interspersed with drought moments, evidenced by low content in sediment water. This species is quite adapted to waterlogged and salinity conditions but is more sensitive to drought. The sugar content was high, especially sucrose, to mediate osmoregulation, guarantee the balance between moments of drought and waterlogging twice a day, and maintain cell turgor under saline stress. The highest FA content was also found in this plant, indicating full metabolic activity. Some β-oxidation has occurred due to the lower concentration of SFA than in Ílhavo. There seems to be no lipid peroxidation, and the plasma membrane is quite fluid.

The presence of *myo*-inositol, glycerol, and tocopherol may be an adaptative antioxidant response for acclimatization. The high content of intermediates of the TCA cycle, like citric, succinic, and malic acid, indicates the cycle’s continuity and probably photosynthesis is not inhibited. However, gluconic and pyroglutamic acids, as well as GABA and proline, were also found in these plants, probably due to osmoregulation acclimatization response but not inhibition of the TCA Cycle. The absence of phenolic acids may be because they are being mobilized for other pathways of synthesis of polar phenolic compounds, since antioxidant molecules were expected under these environmental conditions.

The flood frequency in the São Jacinto salt marsh is the lowest among the sampling locations, and the salinity is lower and similar to Ílhavo. *S. perennis* is subjected to milder sedimentary conditions. The natural physicochemical characteristics of salt marshes increase the sugar content, mostly in stems, as compatible solutes to maintain cellular turgor. These plants show low FA concentration, therefore, are under β-oxidation to face their carbohydrate needs and maintain osmotic potential and cellular metabolism. However, SFA and UFA are not out of balance but acclimating to the unfavorable environmental conditions imposed by the São Jacinto salt marsh sediment. The relatively high content of total sterols increases the stability of cellular membranes and reduces their permeability. Additionally, this plant has some glycerol, mannitol/sorbitol to deal with high salinity as osmolites, and tocopherol, caffeic acid, and ferulic acid to reduce the activity of ROS from salinity and waterlog. The accumulation of pyroglutamic acid, GABA, and proline may indicate inhibition of the TCA cycle. Still, the presence of intermediates compounds in this pathway demonstrates that the cycle continues.

## 4. Conclusions

The lipophilic profile of *S. perennis* and their respective organs, stems, vegetative segments, and fruit segments analyzed for three different sites showed a much greater diversity of compounds than the studies so far. A total of 103 compounds were identified, of which 79 were quantified, including sugars, saturated and unsaturated fatty acids, sterols, alcohols, polyols, waxes, organic acids, and amino acids. The families of compounds that dominated in concentration were sugars and fatty acids for all sampling sites.

The diversity and concentration of compounds in the phytochemical profile varied according to the intensity of environmental stress. It was possible to identify acclimatization responses to more saline and flooded environments, as well as under attack by pathogens. More diverse profiles were found in plants under greater saline and waterlogging stress in Mira, with higher content of saturated and unsaturated fatty acids. In Ílhavo, the lipophilic profile was different than expected for such environmental conditions and led us to believe that the plant is under great stress generated by pathogens. São Jacinto was the salt marsh with the mildest environmental conditions, with the lowest content of fatty acids.

Future studies more focused on identifying the *S. perennis* polar profile would add information concerning the species economic value and could reveal other compounds of interest. Cultivation of plants under controlled environmental conditions of salinity, waterlogging, drought, and subsequent analysis of compounds could provide more accurate information about acclimatization responses.

## Figures and Tables

**Table 1:** Results of Principal Component Analysis (PCA) of Phytochemical family’s data (left) and Environmental data (right). Variables with the highest loadings on the axes in bold type.

Phytochemical Data						Environmental Data		
Variable	PC1	PC2	PC3	PC4	PC5	Variable	PC1	PC2
Saturated fatty acid	0.203	**0.398**	0.150	0.195	0.078	Salinity (‰)	**0.615**	0.190
Saturated fatty amide	0.375	−0.004	−0.057	0.355	0.124	Water content (%)	**−0.624**	−0.131
Saturated fatty ester	**−0.412**	−0.103	0.065	0.028	−0.019	Tidal level (m)	**−0.478**	**0.535**
Monoacylglycerol	0.068	**0.450**	0.092	0.031	0.194	Flood frequency (%)	0.070	**0.813**
Unsaturated fatty acid	−0.177	**0.452**	0.200	0.054	0.173			
Unsaturated fatty amide	0.150	−0.145	0.175	**−0.574**	0.358			
Unsaturated fatty ester	−0.266	−0.244	−0.196	0.269	0.318			
Alcohols	0.366	−0.062	−0.018	−0.231	0.382			
Sugar alcohol	−0.252	0.337	0.346	0.050	0.056			
Sugar	−0.124	−0.354	**0.430**	0.020	0.156			
Alkanes	−0.175	0.090	**−0.469**	−0.052	−0.060			
Saturated fatty alcohol	0.378	0.104	−0.347	0.057	−0.040			
Organic acids	0.164	−0.086	0.247	0.076	**−0.625**			
Phenolic acids	0.089	−0.242	0.111	0.600	0.298			
Sterols	0.312	0.127	−0.366	−0.023	0.153			
% of Variation	28.4	23.3	15.6	0.195	0.078	% of Variation	62.6	37.4

**Table 2:** Mean values ± standard deviation of physicochemical sediment conditions of *Salicornia perennis*. *n* = 5. MHW: mean high water, flooded all year round; MHWN: mean high water neaps, flooded all year round. * Data provided by NMEC-Estuarine and Coastal Modelling.

	Ílhavo	Mira	São Jacinto
Salinity (‰)	23.9 ± 1.17	34.0 ± 0.84	22.5 ± 2.98
Water content (%)	64.27 ± 4.90	32.562 ± 7.88	59.34 ± 2.06
Tidal level *	MHW—1.20 m	MHWN—0.89 m	MHWN—0.90 m
Flood frequency (%) *	70.98 ± 4.95	68.18 ± 8.45	46.85 ± 6.41

**Table 3:** Average (±standard deviations) quantification of sugars (µg/mg), by location, and plant’s organs. fruit = fruiting segments; veg = vegetative segments. NQ = unquantifiable.

	Ílhavo			Mira			São Jacinto		
Sugars	Stem	Fruit	Veg	Stem	Fruit	Veg	Stem	Fruit	Veg
2-Deoxygalactose						0.74 ± 0.53			
D-Ribose	0.24 ± 0.13						NQ		0.37 ± 0.12
Glucose	11.99 ± 1.59	9.05 ± 3.01	25.06 ± 8.95	9.75 ± 2.14	4.96 ± 0.93	19.24 ± 4.71	11.90 ± 3.31	8.19 ± 4.28	24.83 ± 5.09
Sucrose	153.97 ± 43.37	16.56 ± 4.16	42.71 ± 15.95	192.58 ± 42.25	4.86 ± 1.20	32.49 ± 9.79	231.57 ± 70,79	11.82 ± 3.69	45.83 ± 6.28
**Total**	**166.20**	**25.61**	**67.77**	**202.33**	**9.82**	**52.47**	**243.47**	**20.01**	**71.03**

**Table 4:** Average (±standard deviations) quantification of fatty acids and their derivatives (µg/mg), by location, and plant’s organs (Stems; Fruit = fruiting segments; Veg = vegetative segments. NQ = unquantifiable.

	Ílhavo			Mira			São Jacinto		
Compounds	Stem	Fruit	Veg	Stem	Fruit	Veg	Stem	Fruit	Veg
**Saturated fatty acid**									
Arachidic acid	0.24 ± 0.09	0.28 ± 0.08	0.32 ± 0.19	0.21 ± 0.08	0.44 ± 0.12		0.14 ± 0.07	0.09 ± 0.04	0.07 ± 0.03
Behenic acid	1.92 ± 0.97	0.80 ± 0.17	1.17 ± 0.58	1.30 ± 0.31	0.54 ± 0.10	0.38 ± 0.09	1.70 ± 0.12	0.51 ± 0.11	0.32 ± 0.06
Caproic acid		1.47 ± 0.30							
Caprylic acid	NQ	0.18 ± 0.07							
Ceratinic acid	0.16 ± 0.07	0.57 ± 0.12	0.74 ± 0.43		0.47 ± 0.38	0.31 ± 0.08	NQ	0.47 ± 0.12	0.20 ± 0.09
Hexonic acid				0.08 ± 0.04					
Lignoceric acid	0.57 ± 0.16	0.83 ± 0.17	1.06 ± 0.56	0.40 ± 0.31	0.63 ± 0.13	0.58 ± 0.17	0.36 ± 0.19	0.44 ± 0.21	0.20 ± 0.06
Margaric acid	NQ		0.48 ± 0.81	NQ	NQ	NQ		0.30 ± 0.17	
Montanic acid					0.08 ± 0.06	0.09 ± 0.07		NQ	0.05 ± 0.04
Myristic acid	0.15 ± 0.04	0.39 ± 0.10	0.62 ± 0.32	0.17 ± 0.04	0.31 ± 0.04	0.49 ± 0.17	0.17 ± 0.05	0.35 ± 0.10	0.41 ± 0.08
Nonadecanoic acid				0.16 ± 0.21					
Palmitic acid	4.02 ± 0.54	6.88 ± 1.32	8.90 ± 4.43	4.79 ± 0.57	14.14 ± 1.29	5.73 ± 1.09	3.46 ± 0.92	3.49 ± 0.83	3.71 ± 0.61
Pelargonic acid	0.08 ± 0.03		0.15 ± 0.12						
Pentadecanoic acid	NQ			NQ					
Stearic acid	0.77 ± 0.43	1.22 ± 0.33	0.84 ± 0.40	0.74 ± 0.29	1.84 ± 0.41	0.48 ± 0.16	0.54 ± 0.36	0.87 ± 0.30	0.62 ± 0.25
**Total**	**7.91**	**12.62**	**14.28**	**7.85**	**18.45**	**8.06**	**6.37**	**6.52**	**5.58**
**Saturated fatty amide and ester**									
Palmitamide	0.04 ± 0.03								
Stearamide		0.05 ± 0.02	0.36 ± 0.30			0.04 ± 0.02			
Stearyl palmitate				0.61 ± 0.25			0.34 ± 0.16	0.41 ± 0.22	
Cetyl palmitate	0.26 ± 0.07			0.58 ± 0.46	0.39 ± 0.10	0.51 ± 0.08	0.52 ± 0.15	0.60 ± 0.32	0.19 ± 0.07
**Total**	**0.30**	**0.05**	**0.36**	**1.19**	**0.39**	**0.55**	**0.86**	**1.01**	**0.19**
**Monoacylglycerol**									
2-Monopalmitin					0.17 ± 0.13				
Monopalmitin	NQ	NQ	2.54 ± 2.32	NQ	1.07 ± 0.25	1.40 ± 1.05		NQ	0.67 ± 0.45
2-Monolinolenin					0.34 ± 0.14	2.12 ± 0.78		0.25 ± 0.13	1.41 ± 0.92
Monolinolein				0.70 ± 0.35	4.99 ± 0.56		0.59 ± 0.21		
Monolinolenin						1.43 ± 0.32			0.48 ± 0.13
Monostearin	1.06 ± 0.94	0.57 ± 0.53	NQ	0.95 ± 0.84		NQ	NQ		NQ
**Total**	**1.06**	**0.57**	**2.54**	**1.65**	**6.57**	**4.95**	**0.59**	**0.25**	**2.56**
**Unsaturated fatty acid**									
2-Heptenoic acid	NQ	0.16 ± 0.11	NQ						
Linoleic acid	NQ			14.47 ± 2.51	35.87 ± 4.57	3.79 ± 0.85	3.22 ± 1.04	2.70 ± 1.28	1.42 ± 0.62
Linolenic acid						6.83 ± 1.31		1.76 ± 0.80	1.91 ± 0.50
Oleic acid	NQ			NQ	16.78 ± 2.02	NQ	3.57 ± 1.94	NQ	NQ
**Total**	**0**	**0.16**	**0**	**14.47**	**52.65**	**10.62**	**6.79**	**4.46**	**3.33**
**Unsaturated fatty amide and ester**									
Oleamide	20.02 ± 0.49	0.95 ± 0.18	2.08 ± 1.84	1.79 ± 0.55	1.63 ± 0.23	0.99 ± 0.44	1.52 ± 0.69	1.66 ± 0.84	0.99 ± 0.53
Oleyl palmitate						0.14 ± 0.09	0.34 ± 0.28	0.25 ± 0.10	
**Total**	**0**	**0.95**	**2.08**	**1.79**	**1.63**	**1.13**	**1.86**	**1.91**	**0.99**

**Table 5:** Average (±standard deviations) quantification of sterols (µg/mg), by location, and plant’s organs (Stem; Fruit = fruiting segments; Veg = vegetative segments.

	Ílhavo			Mira			São Jacinto		
Compounds	Stem	Fruit	Veg	Stem	Fruit	Veg	Stem	Fruit	Veg
Sitosterol	0.34 ± 0.21			2.34 ± 0.79	2.21 ± 0.94	2.62 ± 0.70	2.30 ± 0.63	3.61 ± 0.39	2.90 ± 0.35
Stigmasterol	0.54 ± 0,27		1.21 ± 0.72	0.71 ± 0.23	1.35 ± 0.12	2.38 ± 0.81	0.76 ± 0.23	2.07 ± 0.29	1.58 ± 0.17
Stigmastanol	0.79 ± 0.17	0.57 ± 0.14	0.93 ± 0.90	0.49 ± 0.26	1.02 ± 0.29	1.02 ± 0.24	0.51 ± 0.20	1.47 ± 0.14	1.01 ± 0.13
**Total**	**1.67**	**0.57**	**2.14**	**3.54**	**4.58**	**6.02**	**3.57**	**7.15**	**5.49**

**Table 6:** Average (±standard deviation) quantification of alcohols and sugar alcohols (µg/mg), by location, and plant’s organs (Stem; Fruit = fruiting segments; Veg = vegetative segments. NQ = unquantifiable.

	Ílhavo			Mira			São Jacinto		
Compounds	Stem	Fruit	Veg	Stem	Fruit	Veg	Stem	Fruit	Veg
**Alcohols**									
18-Methylnonadecanol	0.86 ± 0.17	0.34 ± 0.09	0.65 ± 0.38				0.20 ± 0.17	0.16 ± 0.06	
*gama*-Tocopherol						0.11 ± 0.04			
Tocopherol					0.23 ± 0.06	0.48 ± 0.13		0.06 ± 0.04	0.08 ± 0.05
**Total**	**0.86**	**0.34**	**0.65**	**0**	**0.23**	**0.59**	**0.20**	**0.22**	**0.08**
**Sugar alcohol**									
D-Glucitol				0.03 ± 0.02			0.35 ± 0.19		
Glycerol	1.15 ± 0.34	0.45 ± 0.08	NQ	5.89 ± 1.05	7.86 ± 1.35	1.65 ± 0.71	2.20 ± 0.67	1.60 ± 0.84	0.73 ± 0.45
*Myo*-Inositol	0.05 ± 0.01		NQ	NQ			NQ		
Mannitol/Sorbitol							0.25 ± 0.18		
**Total**	**1.20**	**0.45**		**5.91**	**7.86**	**1.65**	**2.55**	**1.60**	**0.73**

**Table 7:** Average (±standard deviations) quantification of waxes (µg/mg), by location, and plant’s organ (Stem; Fruit = fruiting segments; Veg = vegetative segments). NQ = unquantifiable, * *E*,*E*,*Z*-1,3,12-Nonadecatriene-5,14-diol.

	Ílhavo			Mira			São Jacinto		
Compounds	Stem	Fruit	Veg	Stem	Fruit	Veg	Stem	Fruit	Veg
**Alkanes Total**	**0**	**1.95 ± 0.76**	**0**	**0**	**2.0 ± 1.81**	**0**	**0**	**6.42 ± 0.94**	**0.53 ± 0.40**
**Saturated fatty alcohol**									
22-Methyltetracosanol			0.17 ± 0.15					NQ	0.15 ± 0.07
Behenic alcohol	1.14 ± 0.19	0.83 ± 0.18	1.59 ± 0.97	0.12 ± 0.05		0.30 ± 0.06	0.20 ± 0.17	0.66 ± 0.38	0.41 ± 0.11
Cerylic alcohol	0.55 ± 0.10	1.80 ± 0.37	2.29 ± 1.06	0.48 ± 0.20	1.00 ± 0.12	2.28 ± 0.51	0.19 ± 0.10	1.54 ± 0.40	0.97 ± 0.13
Nonadecatrienediol *					1.43 ± 0.34				
Lignoceric alcohol	0.62 ± 0.09	1.49 ± 0.29	2.54 ± 1.21			1.38 ± 1.11		1.61 ± 0.68	1.37 ± 0.09
Montanyl alcohol	0.35 ± 0.05	0.57 ± 0.12	0.58 ± 0.30	0.43 ± 0.17	0.50 ± 0.07	1.57 ± 0.37	0.14 ± 0.10	0.43 ± 0.10	0.29 ± 0.03
Myricyl alcohol	0.15 ± 0.07			0.44 ± 0.18	0.31 ± 0.11	0.61 ± 0.13		0.24 ± 0.05	0.14 ± 0.03
Palmityl alcohol	0.70 ± 0.10	0.48 ± 0.14	1.26 ± 0.61			0.29 ± 0.13		0.21 ± 0.11	0.50 ± 0.27
Stearyl alcohol	0.68 ± 0.08	0.46 ± 0.19	1.02 ± 0.41			0.29 ± 0.11			0.53 ± 0.15
Oleyl alcohol				NQ		NQ	NQ	NQ	0.12 ± 0.11
**Total**	**4.19**	**5.63**	**9.45**	**1.47**	**3.24**	**6.72**	**0.53**	**4.69**	**4.48**

**Table 8:** Average (±standard deviation) quantification of organic acids (µg/mg), by location, and plant’s organ (Stem; Fruit = fruiting segments; Veg = vegetative segments). NQ = unquantifiable.

	Ílhavo			Mira			São Jacinto		
Compounds	Stem	Fruit	Veg	Stem	Fruit	Veg	Stem	Fruit	Veg
4-Aminobutanoic acid	0.10 ± 0.05			0.49 ± 0.13	0.10 ± 0.06	0.18 ± 0.05	0.24 ± 0.11	0.01 ± 0.00	
Azelaic acid		2.58 ± 0.46							
Butyric acid		0.20 ± 0.13							
Citric acid	0.77 ± 0,24			2.62 ± 0.82	0.07 ± 0.04	1.49 ± 0.53	0.43 ± 0.19		
Cyclopentanoic acid	0.07 ± 0.02	0.15 ± 0.03							
Fumaric acid	0.16 ± 0.07	1.03 ± 0.36	0.44 ± 0.26						
Glucaric acid			0.28 ± 0.17			0.20 ± 0.08		0.04 ± 0.03	0.23 ± 0.09
Gluconic acid/Manoonic acid	0.07 ± 0.06				0.33 ± 0.13	0.06 ± 0.03			
Glyceric acid	0.04 ± 0.02			NQ		0.05 ± 0.03	NQ		NQ
Gulonic acid	NQ		0.73 ± 0.33			0.65 ± 0.39			0.29 ± 0.13
Hydroxycaproic acid		0.06 ± 0.03							
Maleic acid		0.66 ± 0.31	0.23 ± 0.17						
Malic acid	0.47 ± 0.07	0.31 ± 0.09	0.28 ± 0.18	0.57 ± 0.17	0.07 ± 0.03	0.26 ± 0.08	0.19 ± 0.08		0.07 ± 0.03
Octynoic acid	0.33 ± 0.26		0.28 ± 0.26	NQ	NQ	NQ	0.36 ± 0.01	NQ	
Phenylacetic acid				0.57 ± 0.24					
Pimelic acid		0.44 ± 0.19							
Pyroglutamic acid				0.37 ± 0.21		0.08 ± 0.04	0.15 ± 0.09		
Ribonic acid	0.10 ± 0.08		0.22 ± 0.15	0.11 ± 0.06		0.37 ± 0.14	0.46 ± 0.23	0.18 ± 0.06	0.21 ± 0.08
Suberic acid		0.30 ± 0.11	NQ						
Succinic acid	0.15 ± 0.03	0.26 ± 0.09	0.51 ± 0.26	NQ	0.11 ± 0.04	0.38 ± 0.10	NQ	0.03 ± 0.02	0.17 ± 0.04
Benzoic acid		0.05 ± 0.02	0.19 ± 0.14						
Caffeic acid							NQ		
Ferulic acid							0.11 ± 0.05		
**Total**	**2.26**	**6.04**	**3.16**	**4.73**	**0.68**	**3.72**	**1.94**	**0.26**	**0.97**

## Data Availability

Data is contained within the article.

## Supplementary Materials

The following supporting information can be downloaded at: https://www.mdpi.com/article/10.3390/metabo13020280/s1, Table S1: Compounds that could not be quantified for *Salicornia perennis*, by location, and plant’s organ, represented in presence (X) and absence (empty).
