# Supplementary material for: Metabolomic Profile of Salicornia perennis Plant’s Organs under Diverse In Situ Stress: The Ria de Aveiro Salt Marshes Case
Source: Metabolites. 2023 Feb 15;13(2):280. doi: 10.3390/metabo13020280 (PMC9960996; doi:10.3390/metabo13020280)
Supplement: Supplementary file 1 [file metabolites-13-00280-s001.zip › metabolites-2222673-supplementary.pdf]

Supporting Information

Metabolomic Profile of *Salicornia perennis* Plant's Organs under Diverse In Situ Stress: The Ria de Aveiro Salt Marshes Case

Natasha N. Magni 1,2 , Ana C. S. Veríssimo 1 , Helena Silva 2 and Diana C. G. A. Pinto 1,*

1 LAQV-REQUIMTE, Department of Chemistry, Campus Universitário de Santiago, University of Aveiro, 3810-193 Aveiro, Portugal

2 CESAM, Department of Biology, Campus Universitário de Santiago, University of Aveiro, 3810-193 Aveiro, Portugal

* Correspondence: diana@ua.pt; +351-234-401407

Table S1. Compounds that could not be quantified for *Salicornia perennis*, by location, and plant's organ, represented in presence (X) and absence (empty).

	Ílhavo			Mira			São Jacinto		
**Compounds**	**Stem**	**Fruit**	**Veg**	**Stem**	**Fruit**	**Veg**	**Stem**	**Fruit**	**Veg**
**Unsaturated fatty acid**									
10-Undecynoic acid		X	X						
3-Octenoic acid			X		X	X		X	X
Mandenol					X				
Palmitoleic acid				X	X		X		X
Pentenoic acid			X						
Tricosylic acid		X	X						
**Polyphenol**									
Catechine		X			X		X		
**Amina alcohol**									
Ethanolamine							X		
**Amino Acids**									
L-Glutamic acid				X					
L-Isoleucina						X			
L-Proline				X		X	X		
L-Valine				X		X	X		
**Terpene**									
Neophytadiene				X	X	X	X	X	X
Phytol						X		X	X
Phytone		X							
Squalane				X			X		
Perhydrofarnesyl acetone			X						
**Carboxylic acid**									
Pentanedioic acid				X					
**Inorganic**									
Phosphoric acid	X	X	X	X	X	X	X	X	X
Uridine						X			

**Sugar alcohols**				
alpha-Glycerophosphate			X	
**Sugars**				
Erythronic acid	X		X	X
**Alkanes**				
Squalane		X		
Docosane	X			
